# Cortical Structural Connectivity Alterations in Primary Insomnia: Insights from MRI-Based Morphometric Correlation Analysis

**DOI:** 10.1155/2015/817595

**Published:** 2015-10-11

**Authors:** Lu Zhao, Enfeng Wang, Xiaoqi Zhang, Sherif Karama, Budhachandra Khundrakpam, Hongju Zhang, Min Guan, Meiyun Wang, Jingliang Cheng, Dapeng Shi, Alan C. Evans, Yongli Li

**Affiliations:** ^1^McConnell Brain Imaging Centre, Montreal Neurological Institute, McGill University, Montreal, QC, Canada H3A 2B4; ^2^Department of Radiology, Henan Provincial People's Hospital, People's Hospital of Zhengzhou University, Henan 450003, China; ^3^Douglas Mental Health University Institute, McGill University, Montreal, QC, Canada H4H 1R3; ^4^Department of Neurology, Henan Provincial People's Hospital, People's Hospital of Zhengzhou University, Henan 450003, China; ^5^MRI Division, First Affiliated Hospital of Zhengzhou University, Zhengzhou, Henan 450052, China

## Abstract

The etiology and maintenance of insomnia are proposed to be associated with increased cognitive and physiological arousal caused by acute stressors and associated cognitive rumination. A core feature of such hyperarousal theory of insomnia involves increased sensory processing that interferes with the onset and maintenance of sleep. In this work, we collected structural magnetic resonance imaging data from 35 patients with primary insomnia and 35 normal sleepers and applied structural covariance analysis to investigate whether insomnia is associated with disruptions in structural brain networks centered at the sensory regions (primary visual, primary auditory, and olfactory cortex). As expected, insomnia patients showed increased structural covariance in cortical thickness between sensory and motor regions. We also observed trends of increased covariance between sensory regions and the default-mode network, and the salience network regions, and trends of decreased covariance between sensory regions and the frontoparietal working memory network regions, in insomnia patients. The observed changes in structural covariance tended to correlated with poor sleep quality. Our findings support previous functional neuroimaging studies and provide novel insights into variations in brain network configuration that may be involved in the pathophysiology of insomnia.

## 1. Introduction

Chronic insomnia is the most prevalent sleep complaint, which affects about 20% of adult population worldwide [1]. Insomnia is associated with reduced quality of life [2], cognitive impairments [3], physical complaints [4], and poor social functioning [5]. Moreover, chronic insomnia can increase vulnerability for psychiatric disorders [6, 7] and cardiovascular morbidity and mortality [8] and is associated with increased health care consumption [9]. Despite the huge socioeconomic impact of chronic insomnia, by far, its neurobiological correlates have not been well understood. Insomnia can be classified as an independent psychiatric syndrome, known as primary insomnia (PI), and a common comorbidity associated with a variety of physical and psychiatric disorders, known as secondary insomnia [10]. Approximately 25% of the population with chronic insomnia is considered to have PI [11]. Studying chronic PI may allow investigation of the biology of insomnia in a relatively pure condition, independent of influences attributable to any coexisting comorbid medical or psychiatric disorders [12].

In the past two decades, a number of studies using diverse functional neuroimaging techniques have revealed that central nervous system hyperarousal represents a major pathophysiologic pathway in the development and maintenance of insomnia [13–16]. A core feature of the hyperarousal theory of insomnia involves increased sensory processing that interferes with the onset and maintenance of sleep. Patients with PI, relative to healthy sleepers, have been found to show greater high frequency electroencephalographic (EEG) activity in the Beta range around sleep onset [17], during non-rapid eye movement (NREM) sleep [18] and even during normal waking [19]. EEG signals in the Beta range have been known to be a main feature of coherent cortical processing of sensory information. Consistently with such hypothesis, increased intrinsic functional connectivity between sensory-motor regions has been observed in patients with PI based on resting-state functional magnetic resonance imaging (fMRI) [20]. In another resting-state fMRI study [21], patients with PI showed increased functional connectivity of the emotional circuit with the premotor and sensory-motor cortex, which were positively correlated with the severity of insomnia. Therefore, insomnia patients may be in a perpetual cycle of hyperarousal and increased sensitivity to sensory stimulation, which may lead to difficulty in initiating or maintaining sleep.

It has been known that the dynamic emergence of coherent physiological activities that span distinct brain regions making up functional networks is supported by complex structural networks constituted by neuronal elements of the brain [22]. Thus, a question arises: are the pathophysiology of insomnia and the deficits in sensory processing associated with alterations in brain structural connectivity in addition to the aberrant functional connectivity? To explore the answer, we investigated the interregional structural networks for patients with PI and healthy sleepers. Interregional structural networks were constructed using covariance analysis of magnetic resonance imaging- (MRI-) based cortical thickness data. Since axonally connected brain regions are believed to have common trophic, genetic, maturational, and functional interaction effects [23–25], correlations in brain morphology can indicate interregional connectivity [26]. Cortical thickness can reflect in vivo intrinsic characteristics of intracortical morphology including cell size, density, and cell arrangement in a topologically and biologically meaningful way [27]. Structural covariance network (SCN) analysis has extensively been used to investigate brain network alterations during normal development [28, 29] and aging [30] and in diseased populations [31–33].

In the present study, the structural covariance of networks involved in sensory processing was investigated by seeding from the primary visual cortex (PVC), the primary auditory cortex (PAC), and the olfactory cortex (OLF). Based on existing functional neuroimaging findings, we hypothesized that patients with PI would show greater sensory-motor connectivity compared with normal sleepers, and such alteration would be correlated with poor sleep quality.

## 2. Materials and Methods

### 2.1. Participants

This work studied 35 patients with PI (age = 39.3 ± 8.6 years; range 22–55 years; 5 males; 30 females) and 35 healthy controls (age = 34.9 ± 10.7 years; range 23–54 years; 9 males; 26 females), who were recruited at the Neurology Department of the People's Hospital of Zhengzhou University. All participants were right-handed. Diagnosis was performed according to the DSM-IV inclusion criteria for PI [10]. The questionnaire of Pittsburgh Sleep Quality Index (PSQI) [34] was implemented for all participants to evaluate their sleep quality. Please note that PSQI was not used for PI diagnosis and subject classification. All healthy controls reported restorative and satisfactory sleep and had regular sleep habits and obtained PSQI total scores <5. All participants were also screened to ensure that they had no history of chronic neurological or psychiatric disorders, for example, anxiety, and depression, and had never suffered from other sleep disorders. All procedures were approved by the ethics committee of People's Hospital of Zhengzhou University, and written informed consent was obtained from all participants.

### 2.2. MRI Image Acquisition

MRI data acquisition was conducted on a Siemens 3.0 T TrioTim whole-body scanner (Siemens AG, Erlangen, Germany) using a 12-channel array coil. High-resolution structural volumes were obtained using a T1-weighted 3-Dimensional Magnetization Prepared Rapid Acquisition GRE (3D-MPRAGE) sequence (TR = 1,950 ms, TE = 2.30 ms, TI = 900 ms, matrix = 248 × 256, slice thickness = 1 mm, no distance, and FOV = 244 × 252).

### 2.3. MR Image Processing and Cortical Thickness Measurements

The T1-weighted MR images were processed with the CIVET MRI analysis pipeline (version 1.1.12) [35] developed at the Montreal Neurological Institute. First, native MRI images were corrected for intensity nonuniformity using the N3 algorithm [36], and images in the native space were linearly registered into the stereotaxic space [37]. The registered brain volume was segmented into WM, GM, cerebrospinal fluid (CSF), and background [38]. The inner and outer cortical surfaces (each hemispheric surface consisted of 40962 vertices and 81920 triangles) were then automatically extracted using the CLASP algorithm [39]. The obtained cortical surfaces were nonlinearly aligned to a hemisphere-unbiased iterative surface template [40] using a depth-potential function [41] for accurate cross-subject correspondences. Finally, cortical thickness was measured as the Euclidean distance between linked vertices, respectively, on the inner and outer cortical surfaces throughout the cortex with native scaling [27].

### 2.4. Statistical Analysis

The statistical analysis of SCNs was conducted using the SurfStat toolbox (http://www.math.mcgill.ca/keith/surfstat/) for Matlab (R2014a, The Mathworks, Natick, MA, USA).

#### 2.4.1. Cortical Thickness Analysis

Between-group differences in cortical thickness were assessed using vertex-wise analysis of variance across the whole cortex, controlling for age, gender, and the mean cortical thickness. We also explored whether cortical thickness was associated with the sleep quality, measured as PSQI scores, by computing Pearson's correlation coefficients removing the effects of age, gender, and global mean cortical thickness.

#### 2.4.2. Structural Covariance Network Construction

SCNs were constructed by seeding from the primary sensory regions, which were delineated using the Automated Anatomical Labeling (AAL) atlas [42]. These seed regions included the PVC, that is, the bilateral calcarine fissure and surrounding cortex (CAL), the PAC, that is, the bilateral Heschl's gyri (HES), and the bilateral OLF (see Figure S1 in Supplementary Material available online at http://dx.doi.org/10.1155/2015/817595). Prior to the network analysis, a linear regression was performed at every cortical vertex to remove the effects of age and gender. The residuals of this regression were used to substitute for the raw cortical thickness measurements. Then, for each group, the averaged adjusted cortical thickness within each of the seed regions was correlated with all other vertices on the entire cortical surface. Significant correlation was interpreted as connectivity. Following previously reported nomenclature [43], the model fitted for the cortical thickness *T* at a surface point *i* was

(1) $$\begin{array}{c} T_{i}=\beta_{0}+\beta_{1}T_{\text{seed}}+\varepsilon . \end{array}$$

#### 2.4.3. Between-Group Comparisons in Structural Covariance Networks

To compare the structural covariance in the group of patients with PI and in the group of healthy controls, we used linear interaction models, which contained terms for the* seed thickness* and* group*, together with a* seed thickness *×* group* interaction term. We assessed differences in structural covariance between groups by testing the significance of the interaction term at each vertex [43]. The model fitted for the cortical thickness *T* at a surface point *i* was

(2) $$\begin{array}{c} T_{i}=\beta_{0}+\beta_{1}T_{\text{seed}}+\beta_{2}\text{Group}+\beta_{3}\left(\;T_{\text{seed}}*\text{Group}\;\right)+\varepsilon . \end{array}$$

#### 2.4.4. Network Modulations by Sleep Quality

We assessed the modulation of covariance strength by sleep quality that was measured as PSQI score. These models contained terms for* seed thickness* and* PSQI score* as well as a parametric interaction term for* seed thickness* ×* PSQI score*. The model fitted for the cortical thickness *T* at a surface point *i* was

(3) $$\begin{array}{c} T_{i}=\beta_{0}+\beta_{1}T_{\text{seed}}+\beta_{2}\text{PSQI}+\beta_{3}\left(\;T_{\text{seed}}*\text{PSQI}\;\right)+\varepsilon . \end{array}$$

#### 2.4.5. Correction for Multiple Comparisons

Random field theory (RFT) [44] was utilized to correct for multiple comparisons in our surface-based analyses, which controlled the chance of reporting a familywise error (FWE) to *P* < 0.05. To illustrate trends, surface maps were also shown at an uncorrected threshold of *P* < 0.05.

## 3. Results

### 3.1. Demographic Data

Demographic features for the groups of patients with PI and healthy controls are shown in Table 1. The two groups were not significantly different in age and gender proportion (*P* > 0.05, uncorrected). Patients with PI had significantly higher total PSQI score (*P* = 4.52*e* − 23, uncorrected) relative to healthy controls, illustrating that patients had significantly poorer sleep quality.

### 3.2. Cortical Thickness Analysis

**Figure 1:**
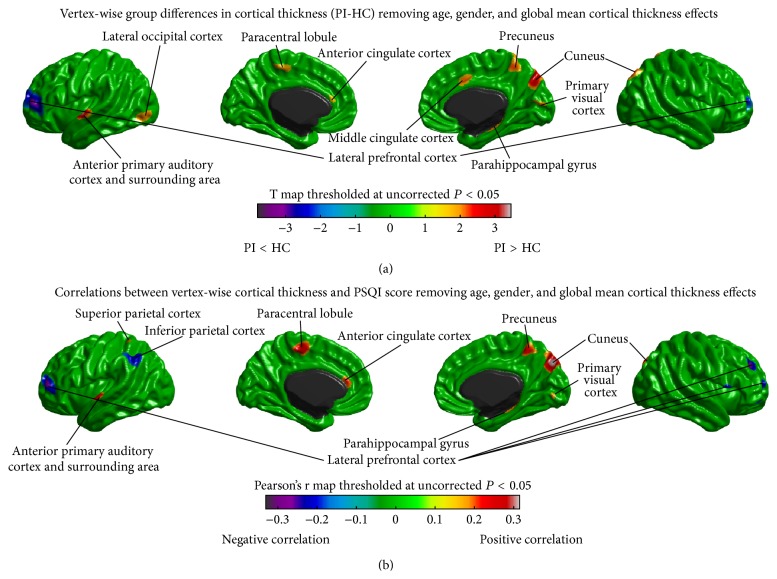
Vertex-wise cortical thickness analysis. (a) Differences in cortical thickness between patients with primary insomnia (PI) and healthy controls (HC), removing effects of age, gender, and global mean cortical thickness. (b) Correlations between vertex-wise cortical thickness and PSQI score removing effects of age, gender, and global mean cortical thickness. No differences and correlations survived the correction for multiple comparisons. Trends (*P* < 0.05, uncorrected) are shown here.

Between-group differences in cortical thickness are illustrated in Figure 1(a). No difference in cortical thickness surpassed the threshold for multiple comparison correction. Analysis of uncorrected differences revealed trends of increased cortical thickness in patients with PI (*P* < 0.05, uncorrected) in the left anterior PAC, the left inferior lateral occipital cortex (LOC), the left the paracentral lobule (PCL), the left anterior cingulate cortex (ACC), the right middle cingulate cortex (MCC), the right cuneus, the right PVC, the right precuneus, and the right parahippocampal gyrus (PHG). In addition, patients with PI also tended to have decreased cortical thickness in the lateral prefrontal cortex (PFC) (*P* < 0.05, uncorrected). Furthermore, correlations between the vertex-wise cortical thickness and the sleep quality (measured as PSQI score, higher PSQI indicates lower sleep quality) are shown in Figure 1(b). The areas showed trends of increased cortical thickness in PI and the left superior parietal cortex (SPC) showed positive correlations (*P* < 0.05, uncorrected) of thickness with PSQI; the areas tended to have decreased thickness in PI and the left inferior parietal cortex (IPC) and the right opercular part of inferior frontal gyrus (IFGoperc) exhibited trends of negative thickness, PSQI correlations (*P* < 0.05, uncorrected).

### 3.3. Structural Covariance Analysis of Visual Networks

**Figure 2:**
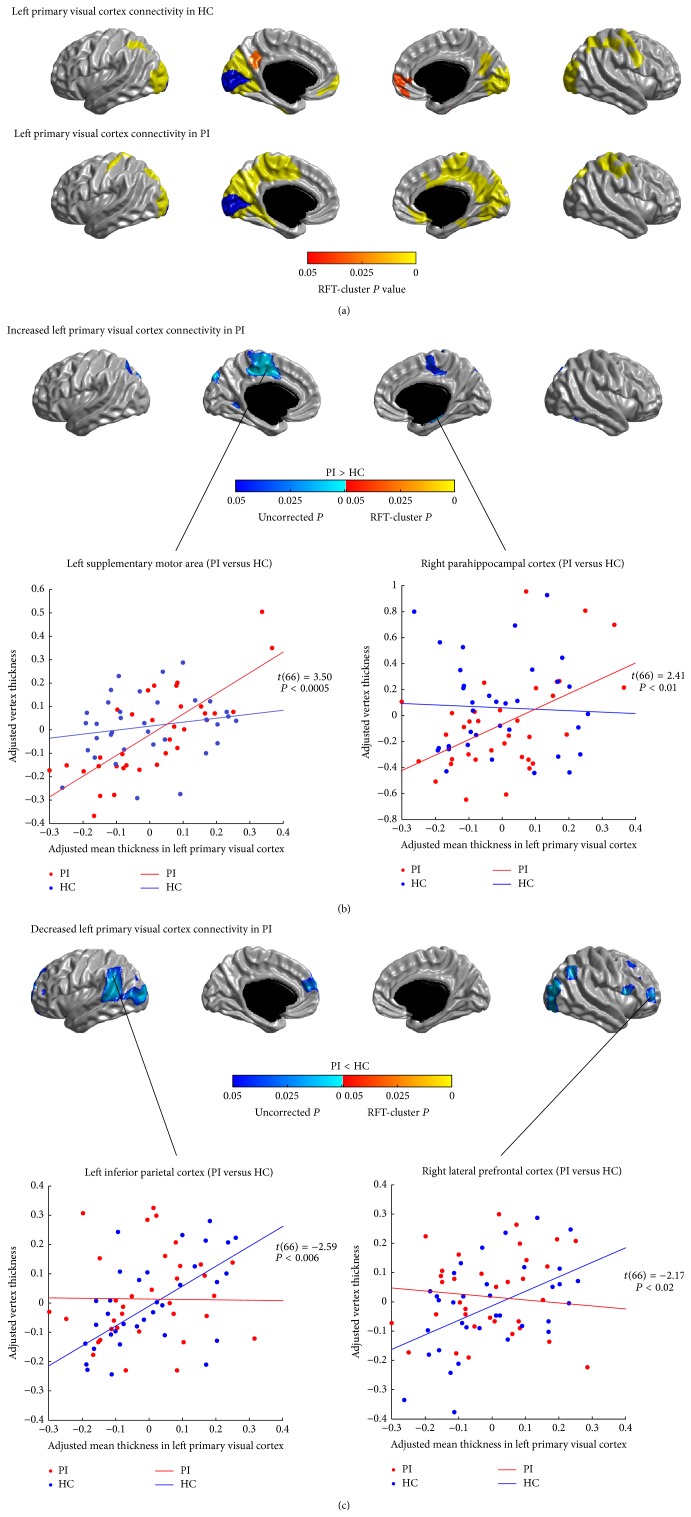
Between-group comparison of structural covariance seeded from the left primary visual cortex (PVC). (a) Structural covariance maps in healthy controls (HC) and patients with primary insomnia (PI). Seed region is colored in blue. (b) Increased left PVC-cortical network covariance in PI. Patients showed trends of increased covariance between the left PVC and the medial motor areas, the left dorsal parietooccipital region, the left cuneus, the right PHG, and the right ITC (*P* < 0.05, uncorrected). (c) Decreased left PVC-cortical network covariance in PI. Patients showed trends of decreased covariance between the left PVC and the lateral PFC, the IPC, the LOC, the left STC, the left dmPFC, and the right IFGoperc (*P* < 0.05, uncorrected). Scatter plots illustrate slope differences in selected vertices, where adjusted cortical thickness values and regression lines are shown in red for patients and in blue for controls.

**Figure 3:**
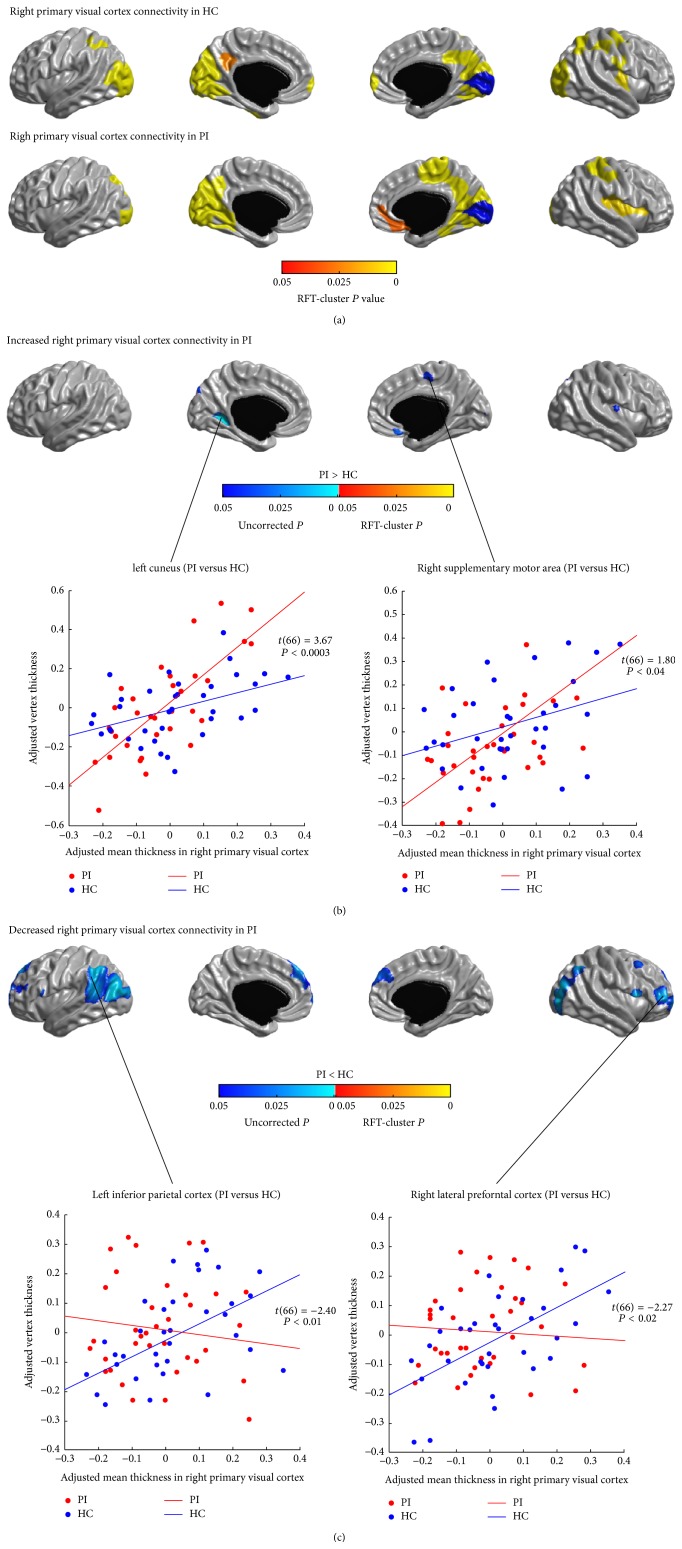
Between-group comparison of structural covariance seeded from the right primary visual cortex (PVC). (a) Structural covariance maps in healthy controls (HC) and patients with primary insomnia (PI). Seed region is colored in blue. (b) Increased right PVC-cortical network covariance in PI. Patients showed trends of increased covariance between the right PVC and the medial motor areas (PCL/SMA) and the left cuneus, the right vmPFC, and the right posterior insula (*P* < 0.05, uncorrected). (c) Decreased right PVC-cortical network covariance in PI. Patients showed trends of decreased covariance between the right PVC and the lateral PFC, the IFGoperc, the IPC, the LOC, the dmPFC, and the left STC (*P* < 0.05, uncorrected). Scatter plots illustrate slope differences in selected vertices, where adjusted cortical thickness values and regression lines are shown in red for patients and in blue for controls.

The SCNs seeded from the left and right PVC (CAL) are illustrated in Figures 2(a) and 3(a), respectively. In healthy controls, the left and right PVC anchored similar structural covariance maps that included the SPC, the right pre- and postcentral gyri, the visual areas (the lateral and medial occipital cortex), the precuneus and posterior cingulate cortex (PCC), and the ventral medial prefrontal cortex (vmPFC) (*P* < 0.05, RFT-cluster corrected). The right PVC additionally encompassed the right posterior insula (*P* < 0.05, RFT-cluster corrected).

In patients with PI, the distribution of significant correlations for PVC (*P* < 0.05, RFT-cluster corrected) was similar to that seen in healthy controls, but more widespread in the medial motor areas (the PCL and the supplementary motor areas (SMA)), the medial temporal cortex (PHG), the left postcentral gyrus, and the right insula and less widespread in the LOC and the left vmPFC.

Figures 2(b), 2(c), 3(b), and 3(c) show the differences in the covariance networks of the PVC between the groups of patients with PI and healthy controls. No difference in the connectivity strength of the left and right PVC surpassed the threshold for multiple comparison correction. Analysis of uncorrected differences revealed trends of increased correlations (*P* < 0.05, uncorrected) between the bilateral PVC and the medial motor areas (PCL/SMA) and the left cuneus, and between the left PVC and the left dorsal parietooccipital region, the right PHG and the right inferior temporal cortex (ITC), and between the right PVC and the right vmPFC and the right posterior insula, in patients with PI. Patients also showed trends of decreased covariance (*P* < 0.05, uncorrected) between the bilateral PVC and the lateral PFC, the IPC, the LOC, the left superior temporal cortex (STC), the dorsal medial prefrontal cortex (dmPFC), and the IFGoperc.

### 3.4. Structural Covariance Analysis of Auditory Networks

**Figure 4:**
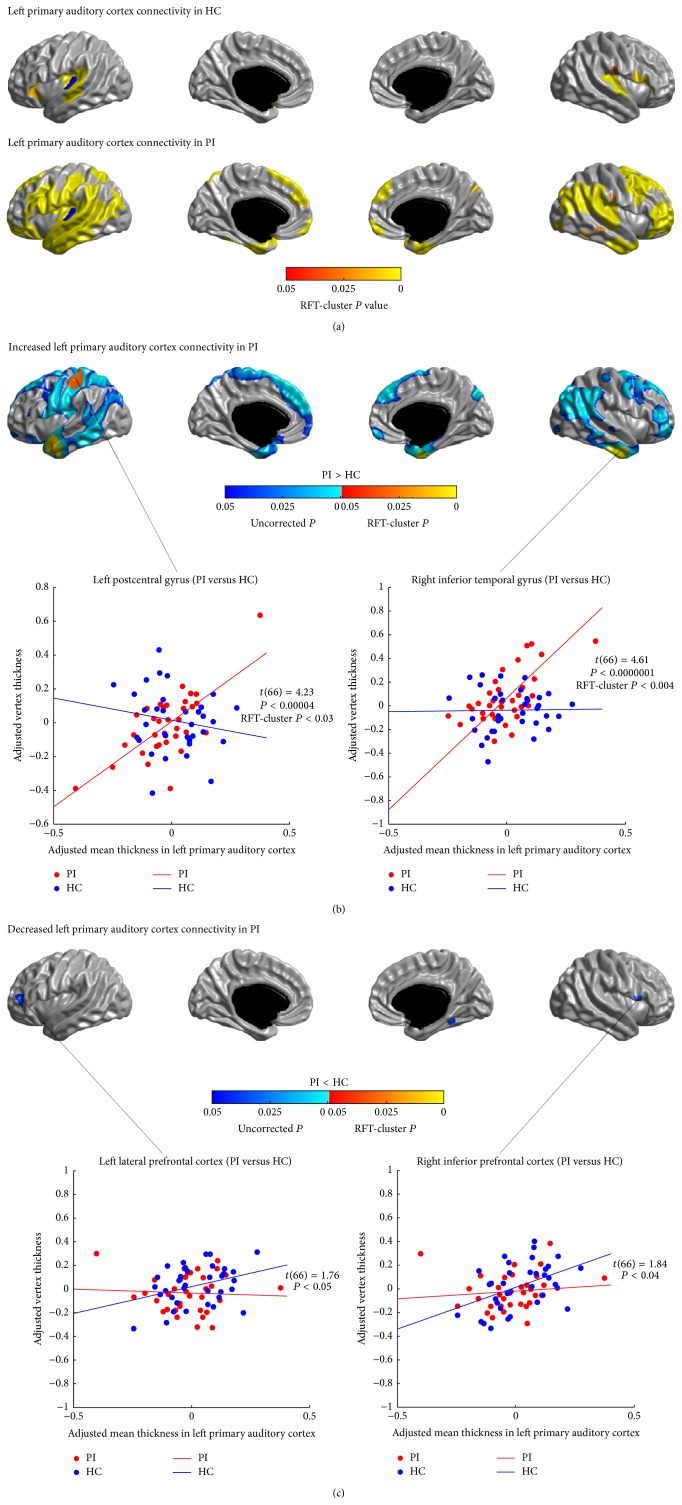
Between-group comparison of structural covariance seeded from the left primary auditory cortex (PAC). (a) Structural covariance maps in healthy controls (HC) and patients with primary insomnia (PI). Seed region is colored in blue. (b) Increased left PAC-cortical network covariance in PI. Patients showed significantly increased correlations of the left PAC with the left postcentral gyrus and the bilateral anterior LTC (*P* < 0.05, RFT-cluster corrected), as well as trends of increased correlations with the dorsal and inferior lateral PFC, the bilateral precentral gyri, the right postcentral gyrus, the LTC, the left posterior insula, the lateral parietal cortex, the medial motor areas (PCL/SMA), the dmPFC, the left OLF, the right vmPFC, and the right superior cuneus (*P* < 0.05, uncorrected). (c) Decreased left PAC-cortical network covariance in PI. Patients showed trends of decreased correlations of the left PAC with the left lateral PFC, the right IFGoperc, and the right FFG (*P* < 0.05, uncorrected). Scatter plots illustrate slope differences in selected vertices, where adjusted cortical thickness values and regression lines are shown in red for patients and in blue for controls.

**Figure 5:**
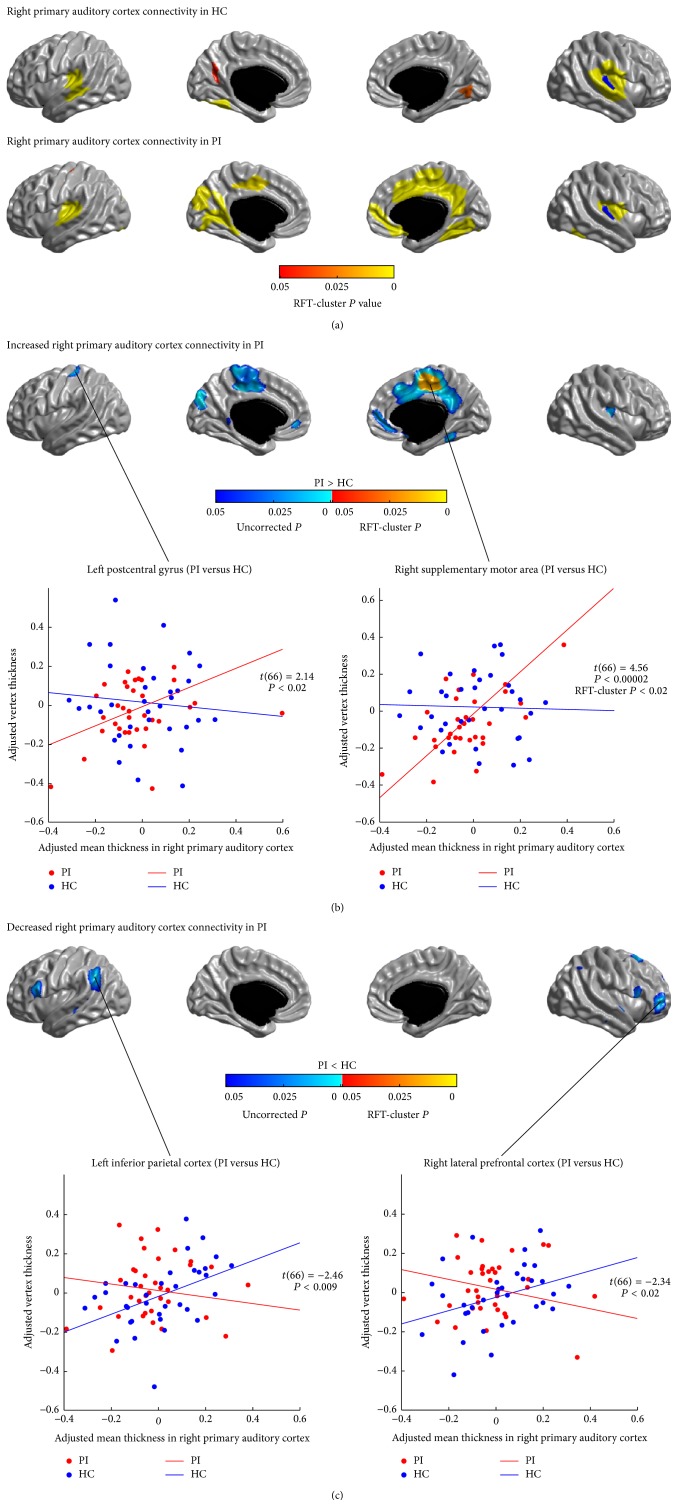
Between-group comparison of structural covariance seeded from the right primary auditory cortex (PAC). (a) Structural covariance maps in healthy controls (HC) and patients with primary insomnia (PI). Seed region is colored in blue. (b) Increased right PAC-cortical network covariance in PI. Patients showed significantly increased correlation with the right medial motor area (PCL/SMA) (*P* < 0.05, RFT-cluster corrected); and trends of increased correlations with the left postcentral gyrus, the left medial motor area (PCL/SMA), the left cuneus, the precuneus/PCC, the vmPFC, the right MCC, the right OLF, the right inferior temporal cortex, and the right posterior insula (*P* < 0.05, uncorrected). (c) Decreased right PAC-cortical network covariance in PI. Patients showed trends of decreased correlations of the right PAC with the IFGoperc, the IPC, the LTC, and the right lateral PFC (*P* < 0.05, uncorrected). Scatter plots illustrate slope differences in selected vertices, where adjusted cortical thickness values and regression lines are shown in red for patients and in blue for controls.

Figures 4(a) and 5(a) show the SCNs seeded from the left and right PAC (HES), respectively. In healthy controls, both the left and right PAC were connected with the bilateral opercular areas (the STC, the posterior insula, and the lower part of the postcentral gyri) (*P* < 0.05, RFT-cluster corrected). The left PAC network additionally included the left and right inferior PFC (*P* < 0.05, RFT-cluster corrected); the right PAC network also included the left and right medial visual areas and the left ITC (*P* < 0.05, RFT-cluster corrected).

The left and right PAC were also connected with the bilateral opercular areas (*P* < 0.05, RFT-cluster corrected) in PI. However, in patients, we observed additional correlations of the left PAC with the lateral PFC, the pre- and postcentral gyri, the lateral temporal and parietal areas, the dmPFC, the vmPFC, and the right superior cuneus; and additional correlations of the right PAC with the left postcentral gyrus, the medial occipital cortex, the medial motor areas (PCL/SMA), the precuneus/PCC, the right dmPFC and vmPFC, and the medial and inferior temporal cortex.

Figures 4(b), 4(c), 5(b), and 5(c) illustrate the differences in the covariance networks of PAC between the groups of patients with PI and healthy controls. In patients, the covariance network of the left PAC showed prominently increased correlations with the left postcentral gyrus and the bilateral anterior lateral temporal cortices (LTC) (*P* < 0.05, RFT-cluster corrected), as well as trends of increased correlations with the dorsal and inferior lateral PFC, the bilateral precentral gyri, the right postcentral gyrus, the LTC, the left posterior insula, the lateral parietal cortex, the medial motor areas (PCL/SMA), the dmPFC, the left OLF, the right vmPFC, and the right superior cuneus (*P* < 0.05, uncorrected). Patients' right PAC network showed significantly increased correlation with the right medial motor area (PCL/SMA) (*P* < 0.05, RFT-cluster corrected), and trends of increased correlations with the left postcentral gyrus, the left medial motor area (PCL/SMA), the left cuneus, the precuneus/PCC, the vmPFC, the right MCC, the right OLF, the right ITC, and the right posterior insula (*P* < 0.05, uncorrected). Furthermore, patients showed trends of decreased correlations (*P* < 0.05, uncorrected) between the bilateral PAC and the lateral PFC and the IFGoperc, and between the left PAC and the right ITC (the fusiform gyrus (FFG)), and between the right PAC and the IPC and the LTC.

### 3.5. Structural Covariance Analysis of Olfactory Networks

**Figure 6:**
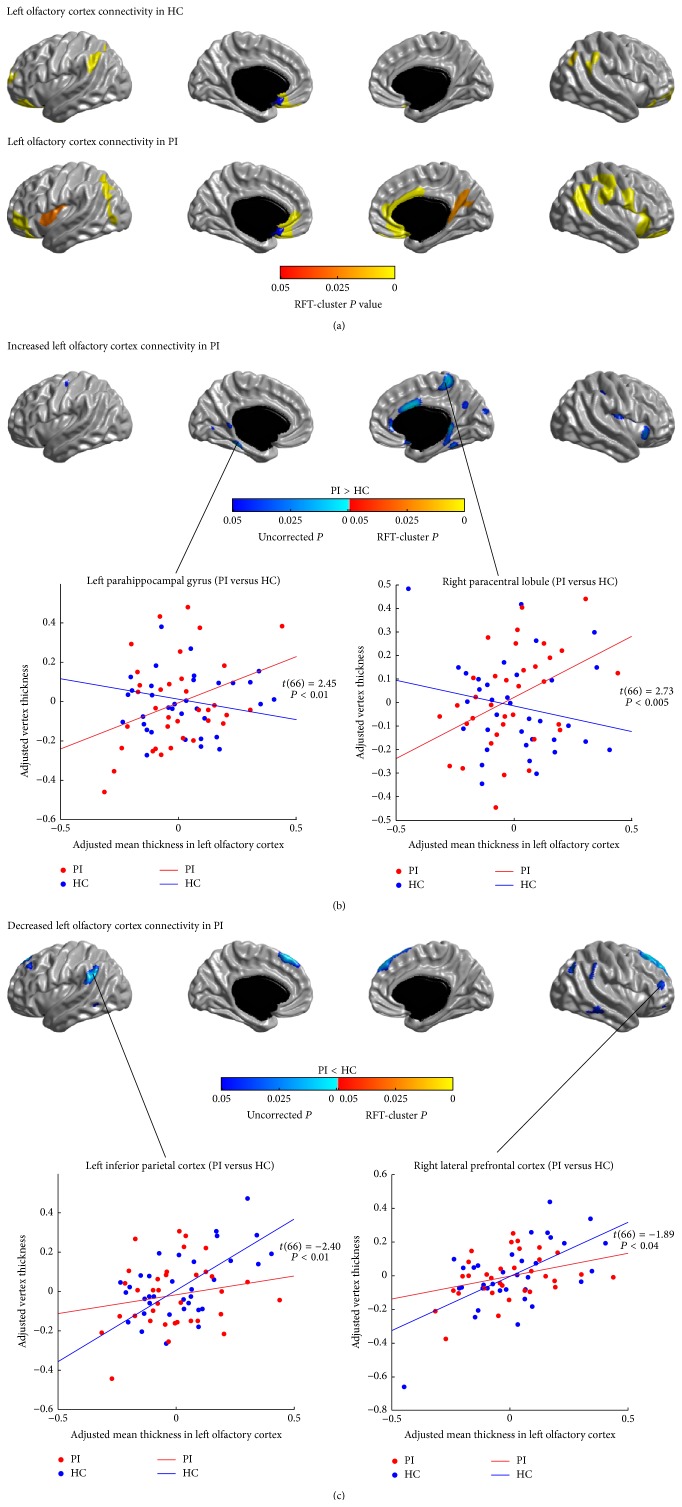
Between-group comparison of structural covariance seeded from the left olfactory cortex (OLF). (a) Structural covariance maps in healthy controls (HC) and patients with primary insomnia (PI). Seed region is colored in blue. (b) Increased left OLF-cortical network covariance in PI. Patients showed trends of increased correlations of the left OLF with the left precentral gyrus, the medial motor areas (PCL/SMA), the PHG, the medial occipital lobes, the right ACC, the right ITC, the lowermost part of the right postcentral gyrus, the right anterior insula, and the bilateral precuenus/PCC (*P* < 0.05, uncorrected). (c) Decreased left OLF-cortical network covariance in PI. Patients showed trends of decreased correlations of the left OLF with the lateral PFC, the IPC, the dmPFC and the LTC (*P* < 0.05, uncorrected). Scatter plots illustrate slope differences in selected vertices, where adjusted cortical thickness values and regression lines are shown in red for patients and in blue for controls.

**Figure 7:**
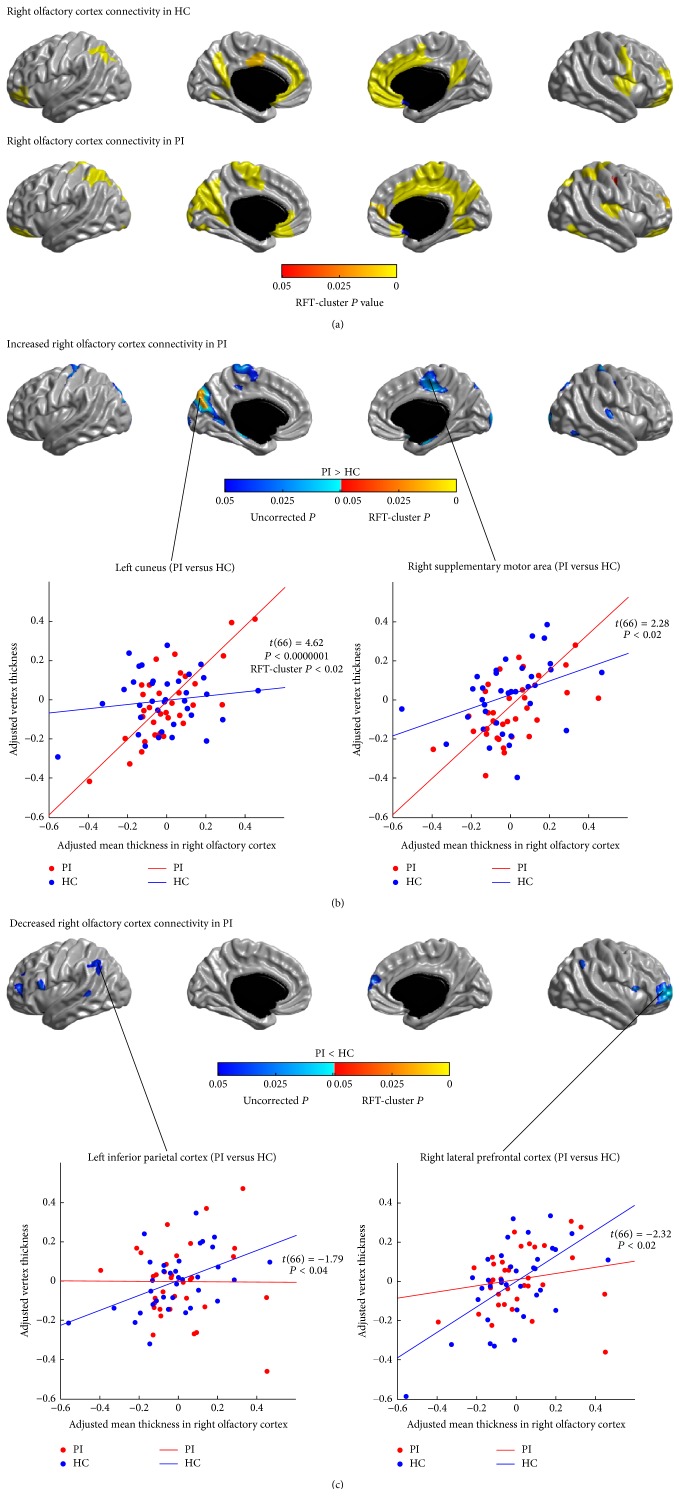
Between-group comparison of structural covariance seeded from the right olfactory cortex (OLF). (a) Structural covariance maps in healthy controls (HC) and patients with primary insomnia (PI). Seed region is colored in blue. (b) Increased right OLF-cortical network covariance in PI. Patients showed significantly increased correlation between the right OLF and the left cuneus (*P* < 0.05, RFT-cluster corrected), and trends of increased correlations with the dorsal central areas, the medial motor areas (PCL/SMA), the PHG, the medial occipital lobes, the dorsal parietooccipital regions, the right inferior occipital gyrus, and the right primary auditory cortex (HES) (*P* < 0.05, uncorrected). (c) Decreased right OLF-cortical network covariance in PI. Patients showed trends of increased correlations between the right OLF and the lateral PFC, the IFGoperc, the IPC, the left LTC, and the right dmPFC (*P* < 0.05, uncorrected). Scatter plots illustrate slope differences in selected vertices, where adjusted cortical thickness values and regression lines are shown in red for patients and in blue for controls.

The SCNs seeded from the left and right OLF are illustrated in Figures 6(a) and 7(a), respectively. In healthy controls, the left and right OLF were mainly connected with the anterior and inferior PFC, the parietal cortex, and the vmPFC (*P* < 0.05, RFT-cluster corrected). The right PVC additionally encompassed the precuneus/PCC, the ACC and the MCC, the dmPFC, the left lingual gyrus, the right precentral gyrus, and the right posterior insula (*P* < 0.05, RFT-cluster corrected).

Similar to controls, patients' OLF SCNs also included the anterior and inferior PFC, the parietal cortex, and the vmPFC (*P* < 0.05, RFT-cluster corrected). However, in PI, the left OLF network showed additional correlations in the bilateral insula, the temporoparietal regions, the ACC, the right MCC, and the lower parts of the right pre- and postcentral gyri. The right OLF network in patients showed more widespread correlations with the dorsal central areas, the superior parietal cortex, the medial motor areas (PCL/SMA), the medial occipital lobes, and the right precuneus, whereas the correlations to the left MCC and the left dmPFC did not reach significance (*P* > 0.05, RFT-cluster corrected).

Figures 6(b), 6(c), 7(b), and 7(c) show the differences in the covariance networks of OLF between the groups of patients with PI and healthy controls. Compared with healthy controls, patients with PI exhibited markedly increased correlation between the right OLF and the left cuneus (*P* < 0.05, RFT-cluster corrected). Both the left and the right OLF networks in PI also showed trends of increased correlations with the dorsal central areas, the medial motor areas (PCL/SMA), the medial temporal cortex (PHG), and the medial occipital lobes (*P* < 0.05, uncorrected). Additionally, in patients, the left OLF network had trends of increased correlations with the right ACC, the right inferior temporal cortex, the lowermost part of the right postcentral gyrus, the right anterior insula, and the bilateral precuneus/PCC (*P* < 0.05, uncorrected); the right OLF network had trends of increased correlations with the dorsal parietooccipital regions, the right inferior occipital gyrus, and the right PAC (HES) (*P* < 0.05, uncorrected). Moreover, patients showed decreased correlations (*P* < 0.05, uncorrected) between the bilateral OLF and the lateral PFC, the IPC, the LTC, and the dmPFC, and between the right OLF and the bilateral IFGoperc.

### 3.6. Relationship between Covariance Strength and Sleep Quality

**Figure 8:**
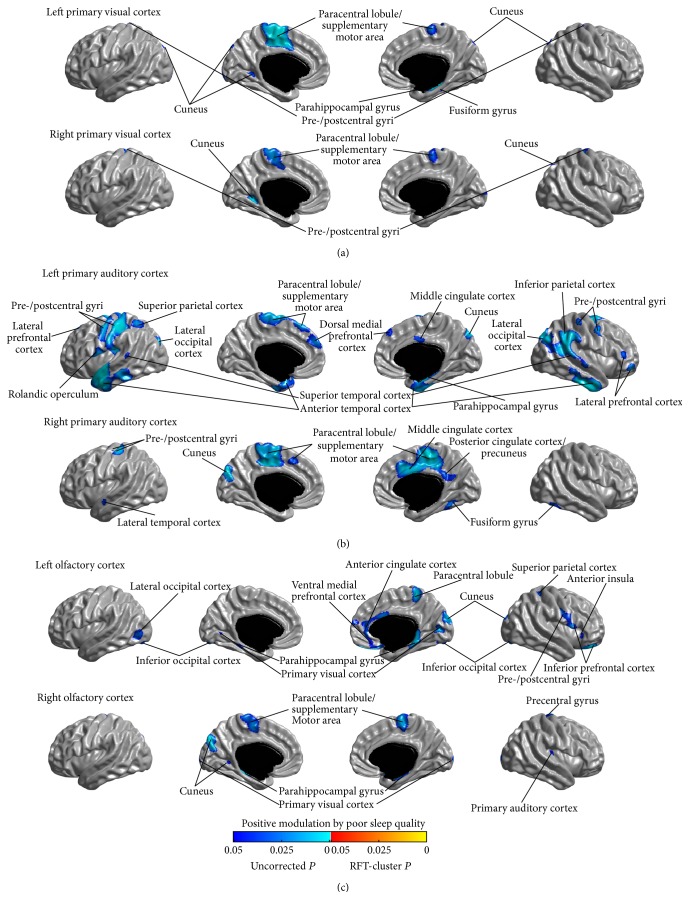
Positive modulations of structural covariance networks by sleep quality, as measured using the Pittsburgh Sleep Quality Index (PSQI) scale. No modulatory effects survived the correction for multiple comparisons. Trends (*P* < 0.05, uncorrected) are shown here.

**Figure 9:**
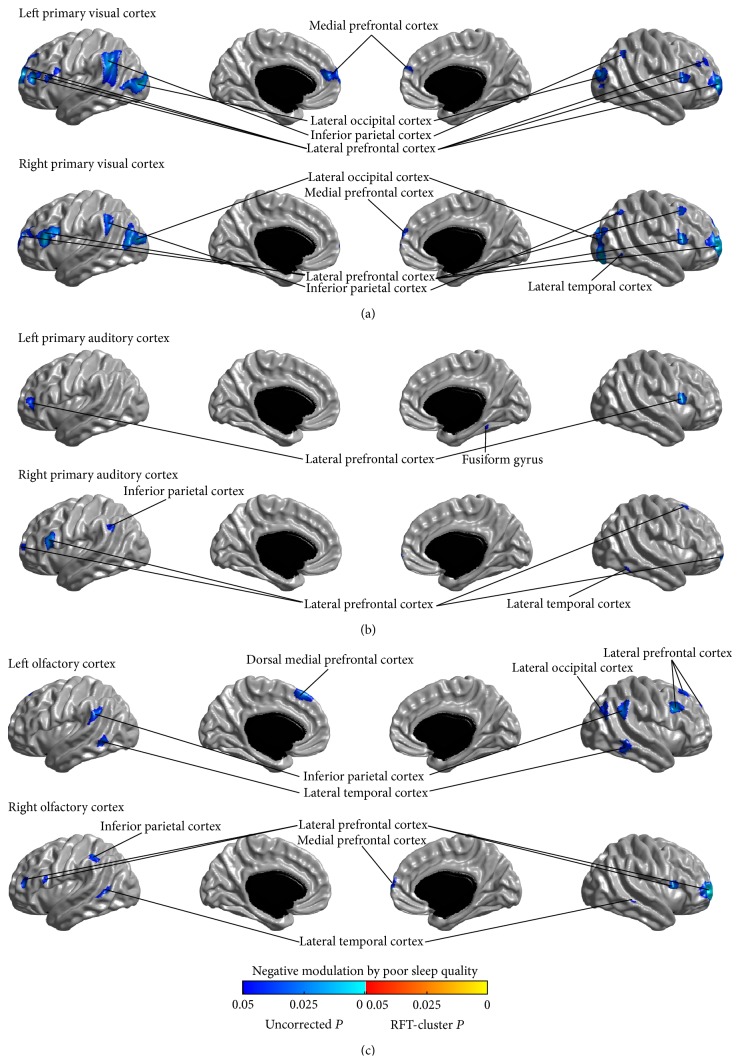
Negative modulations of structural covariance networks by sleep quality, as measured using the Pittsburgh Sleep Quality Index (PSQI) scale. No modulatory effects survived the correction for multiple comparisons. Trends (*P* < 0.05, uncorrected) are shown here.

The modulation of SCNs of the primary sensory regions by the sleep quality was assessed by testing the parametric interaction between PSQI score and seed covariance strength across all participants. We did not observe any significant modulation for all the studied networks (*P* > 0.05, RFT-cluster corrected). Analysis of the uncorrected modulations identified trends of positive and negative modulatory effects of PSQI score on the sensory networks (*P* < 0.05, uncorrected) (positive and negative modulation trends are shown in Figures 8 and 9, resp.); and it is notable that the patterns of the positive and negative modulation trends were, respectively, consistent with the patterns of uncorrected connectivity increase and reduction in PI (Figures 2–7).

## 4. Discussion

This work aimed to test the hypothesis that insomnia would be associated with alterations in networks associated with sensory processing, and sleep quality would be associated with such alterations. We measured and analyzed cortical thickness in groups of patients with PI and normal sleepers using a surface-based method that has been validated and applied to the healthy and diseased brains [27]. Compared with healthy controls, patients with PI tended to have increased cortical thickness, as expected, in sensory (PAC, LOC, PVC, and the cuneus) and medial motor areas (PCL and MCC) and also in some default-mode network (DMN) (the Precuneus and PHG) and salience network (SN) (ACC) regions. Trends of decreased cortical thickness in PI were also observed in the lateral PFC, which plays a key role in working memory [45]. These cortical structural changes were also found to tend to be positively correlated with poor sleep quality. Mapping cortical thickness alterations alone does not provide sufficient information about the underlying pathological mechanisms leading to such changes. On the other hand, since abnormal interactions between cortical regions may be related to the observed neuroanatomical variations [26], we studied the structural covariance connectivity between the primary sensory regions and the neocortex. Compared with healthy controls, patients with PI showed significantly increased covariance or trends of increase in covariance between the seeds of primary sensory regions (PVC, PAC, and OLF) and the motor regions (PCL/SMA, the pre- and postcentral gyri), and between the sensory regions (PAC, OLF, and the cuneus), and between the sensory regions and DMN (the precuneus/PCC, vmPFC, and the anterior LTC and PHG) and SN regions (ACC and the insula). In addition, patients with PI also showed trends of decreased structural covariance between the seeds of primary sensory regions and the frontoparietal working memory network (lateral PFC, IFGoperc, and IPC). Patterns of trends of positive and negative modulatory effects of poor sleep quality on the SCNs in all the participants were greatly in line with the between-group differences in the corresponding networks. These findings suggest that insomnia might be related to underlying increase in brain network integration encompassing the sensory to motor networks, the DMN and the SN, and decrease in brain networks integration of the sensory regions to the frontoparietal working memory network.

SCN analysis relies on the assumption that related regions covary in brain morphology as a result of mutually trophic influences [46] or from common experience-related plasticity [47, 48]. Furthermore, several existing studies have demonstrated a link between the pattern of structural covariance and the architecture of the intrinsic functional networks [49–51]. Therefore, regions that are associated in cortical thickness may also be a part of the same functional network. In this work, the SCNs were seeded from the key regions involved in sensory processing: the PVC, PAC, and OLF. In healthy sleepers, the SCN of PVC mainly included the superior parietal regions, the pre- and postcentral gyri, the visual areas, the PCC/precuneus, and the vmPFC; the SCN of PAC primarily included the bilateral opercular areas (the superior temporal gyri, the posterior insula, and the lower part of the postcentral gyri); the SCN of OLF predominantly included the anterior and inferior PFC, the parietal cortex, and the vmPFC. These network patterns are in overall agreement with data from previous SCN studies [28, 30] and functional connectivity mapping in normal populations [52–55]. Considering the PI-related cortical thickness alterations in the regions showing abnormal structural covariance, we could speculate that the increased or decreased connectivity may reflect a correlative or inverse cortical thickness changes due to enhanced mutual or inhibitory interregional trophic/functional relationship, as affected by the pathological process of PI.

In the current study, compared with the healthy controls, the primary alterations in SCNs in patients with PI were the increased covariance between the sensory and motor regions. Significantly increased covariance was found between the left PAC and the left primary somatosensory cortex (the postcentral gyrus), and between the right PAC and the right medial motor area (PCL/SMA), and between the right OLF and the left cuneus (*P* < 0.05, RFT-cluster corrected). In addition, we also found trends of increased covariance of PVC with the medial motor area (PCL/SMA) and the left cuneus, and of PAC with the primary motor and somatosensory cortex (the pre- and postcentral gyri), the medial motor area (PCL/SMA), the cuneus visual cortex, and the OLF, and of OLF with the primary motor and somatosensory cortex (the pre- and postcentral gyri), the medial motor area (PCL/SMA), the dorsal visual cortex, and the right PAC. The findings are well in line with data from previous functional connectivity analysis. Killgore and colleagues examined resting-state functional connectivity differences between individuals with or without insomnia-related sleep problems [20]. They observed that difficulty in falling asleep was associated with increased functional connectivity between cuneus and other sensory regions such as the PAC and OLF, and the medial motor area (SMA), and between the PAC and the medial motor area (SMA); and problems with sleep maintenance were associated with greater connectivity between the cuneus and the OLF. Their findings indicated that stimulation of one sensory modality might be associated with increased activation of other sensory and motor regions in insomnia. Therefore, greater connectivity among the sensory and motor regions in insomnia may be conceivably associated with sustained arousal and enhance unwanted sensory awareness, leading to difficulty in sleep initialization or maintenance.

Furthermore, patients with PI also showed increased structural covariance between the sensory regions and the DMN regions, such as the precuneus/PCC, the vmPFC, the anterior LTC, and the PHG. In particular, the increased structural covariance between the right PAC and the bilateral anterior LTC survived correction for multiple comparisons (*P* < 0.05, RFT-cluster corrected). Although there is no full understanding about the functions inherent to DMN, in general, DMN concerns the neural network that is active when the individual is not focused on an external task mobilizing the explicit attentional resources and the brain is at wakeful rest [56]. Studies using resting-state paradigms have revealed that DMN is involved in internal modes of cognition, such as mind wandering, recovery of past memories, planning/projection of future events, and consideration of others' perspectives [57]. Dysfunction in DMN has also been related to the neuropathophysiology of insomnia [58]. Drummond et al. [59] investigated the neural correlates of working memory performance in PI, and found that, during behavioral performance, patients with PI failed to deactivate DMN regions compared to good sleepers. Hasler et al. [60] examined the functional connectivity within DMN in patients with PI and good sleepers in different times of the day and found that both groups were not distinct regarding functional connectivity in the morning period after waking up; however, the patient group showed higher DMN connectivity at evening, during NREM sleep, and after sleep restriction. Therefore, the increased structural covariance between the sensory and DMN regions might be related to a high level of arousal in sensory and DMN regions during the day which tends to persist at night and during sleep stages [13]. Alternatively, such structural covariance increase in PI might also be related to pronounced activation of sensory processing and self-referential processes at bedtime or in the absence of tasks/external stimuli [61].

Patients with PI also tended to have increased structural covariance of the sensory regions, especially the left OLF, with the ACC and the insula that are nodes within SN. SN is thought to recruit relevant brain regions for the processing of sensory information and integrate processed sensory data with visceral, autonomic, and hedonic signals in order to guide behavior [62, 63]. Although the role of SN in the mechanism of insomnia is unclear, increased resting-state functional connectivity in SN has been reported in normal subjects after sleep deprivation [64] and patients with PI [65]. Therefore, the trend of increased structural covariance between sensory and SN regions might be associated with the aberrant neural activity in these regions induced by sleep loss.

Another finding in the current study is that patients with PI showed trends of decreased connectivity of the seeds of primary sensory regions with the frontoparietal working memory network consisting of the lateral PFC, the IFGoperc, and the IPC [45]. Such connectivity decrease is in line with the trends of increased cortical thickness in the sensory regions and the trends of decreased cortical thickness in these frontoparietal working memory network regions in PI, observed in the cortical thickness analysis. In Drummond and colleagues' study investigating the neural correlates of working memory performance in PI [59], compared with good sleepers, patients with PI showed reduced activation of the frontoparietal working memory network and specifically reduced modulation of the lateral PFC with increasing task difficulty, in addition to their failure to deactivate DMN regions. Therefore, our results further demonstrated the impairment of the frontoparietal working memory network in PI, which might account for the reported decline of working memory capacity in sleep disorders [66]. Trends of decreased connectivity with the dmPFC were also observed in the networks of the PVC and OLF. Damage of the dmPFC has been associated with insomnia and was thought to disrupt the propagation of sleep slow waves along the insula-cingulate corridor, resulting in difficulty initiating or maintaining sleep [67]. In this study, we were unable to test this hypothesis, which requires EEG sleep recording. In addition, trends of decreased connectivity between the sensory regions and the LOC, the LTC and the ITC were also found in PI. However, no PI-related structural or functional changes in these regions were found in this work or previous studies. Therefore, it is unclear whether and how the trends of decreased connectivity in these regions are related to the pathology of PI. Furthermore, we observed an exception that the left PAC showed trends of increased connectivity with the right lateral PFC, the right IPC, the dmPFC, and the LTC in patients with PI. This may suggest a complementary mechanism in patients with PI to compensate for the decreased connectivity within other SCNs.

We assessed the relationship between the structural covariance and sleep quality by testing the parametric interaction between the PSQI score and the structural covariance strength from seed regions across all participants. Although all modulation of structural covariance by PSQI did not survive multiple comparison correction, analysis of the uncorrected modulations found trends of positive and negative modulatory effects of PSQI score on the sensory networks; and it is notable that the patterns of the positive and negative modulation trends were, respectively, consistent with the patterns of uncorrected connectivity increase and reduction in PI. This further illustrated that the structural covariance alterations in patients with PI observed in this work may be related to the pathophysiology of insomnia.

In this work, we noted some asymmetries between the SCN alteration maps of the left and right sensory regions, for example, the increased structural covariance maps of the left and right PAC. Although, by far, there is no clear evidence about asymmetric effects of insomnia on the brain, previous studies have also reported lateralized patterns of brain structural alterations in PI [67–69]. It is plausible that the asymmetric structural changes may be related to the abnormal functional asymmetries in patients with PI during night or sleep [70, 71]. In addition, we also noted that only the SCNs of the primary auditory and olfactory regions showed statistically significant alterations in patients with PI (survived in the multiple comparison correction), but the SCNs of the primary visual cortex. This might indicate that the sleep deprivation of the patients may be mainly related to the heightened arousal induced by auditory or odor stimuli rather than visual stimuli, when they are in the bed. In line with this, previous study examining event-related potentials during sleep reported that patients with insomnia showed reduced sensory gating of auditory stimuli compared with normal sleepers [72]. However, specific functional study is needed to verify this hypothesis.

There are limitations of the current study that should be addressed in future investigations. First, most present results did not survive the correction for multiple comparisons, which might be caused by the relatively small sample size. It is desirable to increase the sample size in the future work to increase the statistical power. Second, although our findings in structural covariance were qualitatively well in line with the data from previous functional connectivity analyses, it is unclear about the quantitative similarity between the observed SCNs and the functional connectivity networks derived from fMRI data. There is still lack of systematic comparison of structural covariance and functional connectivity networks. It should be cautious to generalize the findings of SCNs in PI to other networks. Combinations of multimodal neuroimaging approaches should yield a more comprehensive understanding of how abnormalities in the brain network architecture are associated with functional deficits. Third, the structural network was group-based so that we could not explore the network organization for individual subjects. Furthermore, subcortical regions were not included in this study, as this study concentrated on the structural covariance in cortical thickness. It is desirable to extend the study to subcortical regions to explore their contributions to the disruptions of the SCNs in PI. Some existing studies, that is, [73], attempted to correlate the volume of certain subcortical regions with the cortical thickness across the cortex or in specific cortical regions; however, it might be questionable to correlate structural metrics at distinct exponential scales. Comparable subcortical structural measurements to cortical thickness are expected for such analysis. Finally, utilizing other network analysis methods in the future work, for example, graph theory analysis to assess the topological patterns of the large-scale brain network organization [22], could enhance the understanding of the brain network organization in insomnia.

In summary, we, for the first time, used a seed-based SCN mapping approach to investigate large-scale structural networks in PI. The results demonstrated increased covariance in cortical thickness between sensory and motor regions, and likely between sensory regions and DMN and SN regions, and trends of decreased connectivity between sensory regions and the frontoparietal working memory network regions, in patients with PI. In addition, the observed alterations in structural covariance tended to be correlated with poor sleep quality. Our findings support the aberrant functional activation and connectivity in insomnia reported by previous functional neuroimaging based studies and also provide novel insights into variations in brain network configuration that may be involved in the pathophysiology of insomnia.

## Supplementary Material

Supplementary Figure S1 illustrates the anatomical locations of the seed regions for structural covariance network construction, which were delineated using the AAL atlas.

## Figures and Tables

**Table 1:** Demographic data of patients with primary insomnia (PI) and healthy controls (HC).

	PI	HC	*P* value
Number of subjects	35	35	
Gender (male/female)	5/35	9/26	0.23^a^
Age in years (M, SD)	39.3, 8.6	34.9, 10.7	0.067^b^
Total PQSI (M, SD)	12.57, 3.93	2.26, 1.36	4.52*e* − 23^c^

M = mean, SD = standard deviation, and PQSI = Pittsburgh Sleep Quality Index.

^a^The *P* value was obtained with a Chi-square test.

^b^The *P* value was obtained with a two-tailed two-sample *t*-test.

^c^The *P* value was obtained with a one-tailed two-sample *t*-test.
