# Supplementary material for: Cortical Structural Connectivity Alterations in Primary Insomnia: Insights from MRI-Based Morphometric Correlation Analysis
Source: Biomed Res Int. 2015 Oct 11;2015:817595. doi: 10.1155/2015/817595 (PMC4619857; doi:10.1155/2015/817595)
Supplement: Supplementary file 1 — Supplementary Figure S1 illustrates the anatomical locations of the seed regions for structural covariance network construction, which were delineated using the AAL atlas. [file 817595.f1.pdf]

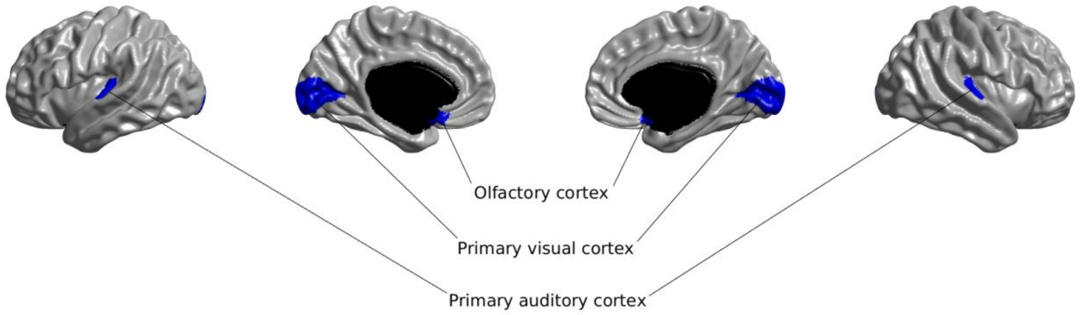

Supplementary Figure S1: Seed regions for structural covariance network construction (the bilateral primary visual cortex (calcarine fissure and surrounding cortex), the bilateral primary auditory cortex (heschl's gyri) and the bilateral olfactory cortex) delineated using the AAL atlas.
